# In-office fluoroscopy is an underutilized tool in the work-up of the bariatric and foregut patient

**DOI:** 10.1007/s00423-025-03842-1

**Published:** 2025-10-27

**Authors:** Terive Duperier, Pial Hope, Samik Patel, Alex Tse, Jordan Purewal, Richard Englehardt

**Affiliations:** The University of Texas Health Science Center at Houston, Houston, USA

**Keywords:** Bariatric surgery, Foregut surgery, Fluoroscopy, Sleeve gastrectomy, Gastric bypass

## Abstract

**Background:**

In-office fluoroscopy can be a useful tool for foregut and bariatric surgeons. It can be used to evaluate patients before and after surgery. Fluoroscopy provides a platform to teach patients about their anatomy and even be used to modify behavior. Based on our clinical experience, IOF appears to provide valuable insights in selected patient populations where conventional diagnostics are inconclusive Xu (BJR|case Rep 3:1-20160076, 2017).

Our experience with fluoroscopy began with the popularity of adjustable gastric bands. Our practice does not advocate for the blind access of band ports. Because there are multiple types of bands made by multiple companies we find that the blind accessing of ports and thus the blind guessing of the amount of fluid to put in or take out of a band is unacceptable. For years, we utilized fluoroscopy for our band patients to accurately and safely adjust their bands to create the desired amount of restriction [2]. As the band fill frequency and popularity has waned, we fortuitously found that fluoroscopy was helpful in other scenarios.

**Methods:**

Between January 2019 and December 2024, patients presenting to our bariatric and foregut surgery clinic underwent IOF based on clinical indications, including postoperative symptoms, evaluation of prior surgical anatomy, or preoperative assessment for GERD. All procedures were performed using a GE Healthcare OEC One C-arm and interpreted by the attending surgeon.

We present **11 representive cases** to illustrate the clinical utility of IOF. Patient consent for use of de-identified imaging and clinical data was obtained.

## Clinical vignettes overview

**Table:** 

Case	Diagnosis	IOF findings	Intervention	Outcome
1	Gastric band obstruction	Delayed outflow, "muffin top" pouching	Band deflation	Immediate symptom resolution
2	Paraesophageal hernia	Large intrathoracic stomach, delayed emptying	Repair + Toupet fundoplication	Smooth gastric drainage
3	Achalasia post-sleeve	Bird's beak sign	Robotic Heller myotomy	Symptom resolution
4	LINX dysfunction	Severe dysmotility, functional obstruction	LINX explant + RNYGB	Resolved dysphagia
5	Sleeve kinking	"Pac-Man" sign, mechanical obstruction	Adhesiolysis	Improved PO intake
6	Gastroparesis post-sleeve	Delayed sleeve emptying	Pyloromyotomy	Improved oral tolerance
7	Enlarged gastric pouch	Right-corner GJ outlet, slow drainage	Partial pouch resection	Resolved symptoms
8	Candy Cane syndrome	Afferent limb preferential filling	Resection of afferent limb	Symptom resolution
9	GJ stricture	Obstructed pouch emptying	GJ revision	Improved PO tolerance
10	Achalasia with retained fundus	Bird's beak, delayed emptying	Heller myotomy	Normal esophageal clearance
11	Sleeve "p-trap"	Delayed contrast clearance at angulation	RNYGB conversion	Resolved reflux and vomiting

## Introduction

The increasing prevalence of bariatric and foregut surgeries [3] has brought diagnostic challenges associated with post-operative anatomy and complications. While radiologic studies and endoscopy are standard, they are often limited by static imaging or non-dynamic evaluation.

**In-office fluoroscopy (IOF)** provides immediate, dynamic visualization of the gastrointestinal tract under the direct supervision of the treating surgeon. It is cost-effective, safe, and offers an unparalleled opportunity for real-time functional assessment.

This educational review aims to illustrate the value of IOF through selected clinical vignettes, emphasizing its role not only in diagnosis but also in patient education and management.

## Case 1: Adjustable gastric band and esophageal motility

**Fig. 1:**
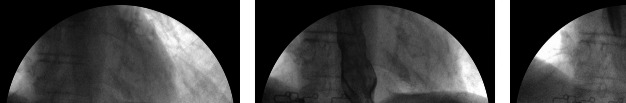
(left): no outflow of contrast through the level of the band on initial evaluation. (middle): only small amount of contrast passed through after a few minutes. (right): Improvement of emptying after removal 2.5 ml of fluid from the band

65 year-old female had a laparoscopic band placed nearly 20 years ago and had not been seen in the office for more than 13 years. The patient had been doing well until recently, when they developed nausea, emesis and food intolerance. They would have 4–5 episodes of emesis per week. The patient reported significant interference with daily functioning. We performed an in-office fluroscopy (refer to Fig. 1 (left)) which showed no outflow of contrast through the level of the band; there was the beginning of a “muffin top” of contrast where the pouch hangs over the band. After several minutes of observation, only a sliver of contrast flowed through the band outlet (refer to Fig. 1 (middle)). Also note that the position of the band is nearly horizontal and this can be indicative of future problems.

We removed 2.5 mL of fluid from the band under fluoroscopic guidance, which led to immediate resolution of the patient’s symptoms. Repeat UGI showed contrast emptying easily through the band and the patient felt much improved (refer to Fig. 1 (right)). The patient was immediately able to tolerate liquids without nausea, vomiting, or reflux symptoms. The patient returned to work the same day and her symptoms resolved after the intervention.

IOF has taught us that there appears to be an age-related component to esophageal motility that has never been thoroughly studied. Here we have a patient who has not had issues for more than 15 years. The patient had a band placed in her 30 s when her esophageal motility was normal. With time and age there appears to be some mild esophageal motility coordination and strength that diminishes. As such, age-related decline in esophageal motility may have contributed to the patient’s symptoms. When surgeons were initially proctored in adjustable gastric band placement, this phenomenon was not appreciated or discussed. In our surgical practice we have observed this change. This is now the reason many bands are removed and why the band has ‘failed’ in many cases. Patients like this may not necessarily need their band removed—they need it adjusted under direct vision. We see this manifest in the office frequently when patients present and state that they are having more “reflux” and oral intolerance with their bands despite not having had an adjustment for years. The band has not gotten tighter—the distal esophagus appears to have become less coordinated and weaker, leading to the patient’s symptoms. With IOF a small amount of fluid is precisely removed to achieve the desired tightness of the band, especially as patients get older.

When patients insist on having a band placed, when patients are interested in magnetic sphincter augmentation, or when patients are having foregut complaints, IOF allows for the evaluation of esophageal motility and its appropriateness for surgery.

## Case 2: GERD and paraesophageal hernia

**Fig. 2:**
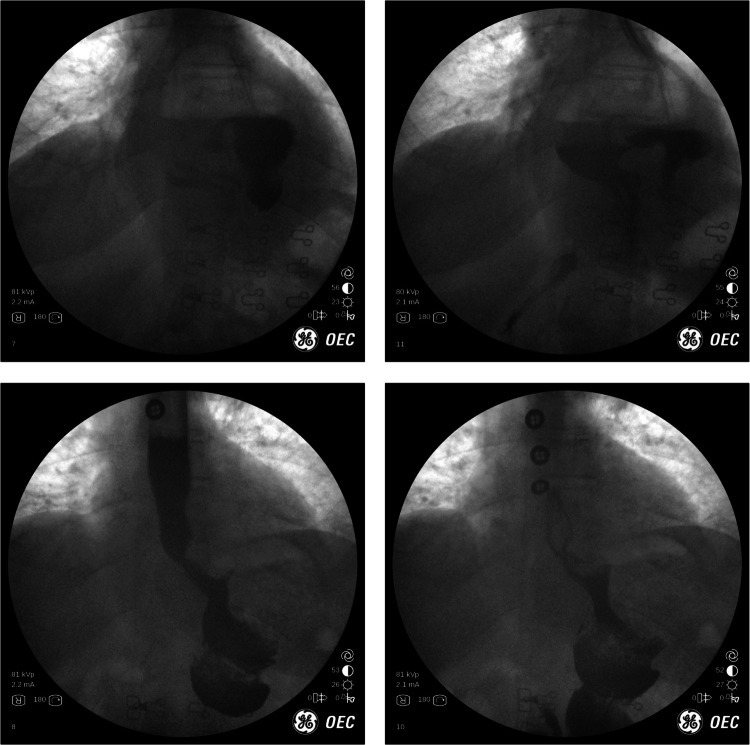
Top: Pre-op UGI images. Bottom: Post-op UGI images

53 year-old female presented to our clinic with a history of GERD and a known hiatal hernia for several years. They were having both typical and atypical symptoms of GERD. An upper GI was performed which showed a large paraesophageal hernia with > 50% of the stomach within the chest.The pre-op UGI image to the left (Fig. 2) demonstrates large paraesophageal hernia with some contrast sitting in the stomach above the diaphragm. As demonstrated by the image to the right (Fig. 2), the contrast then slowly emptied into the duodenum after several minutes. Given the size of the paraesophageal hernia and some concerns about possible underlying gastroparesis from chronically herniated stomach, we opted to perform a paraesophageal hernia repair with Toupet fundoplication. The patient recovered without uneventfully from the surgery. Postoperatively, IOF demonstrates the stomach below the diaphragm with contrast emptying smoothly into the stomach and duodenum.

While IOF did not add any direct benefit to the diagnosis, it did offer some important things. Some patients with long standing large paraesophageal hernias can have underlying gastroparesis. This cannot be diagnosed with endoscopy or CT scan. The best way to exclude it would be from a gastric emptying study. However, in our experience when a patient demonstrates normal gastric motility on UGI the gastric emptying study is not mandatory. By doing this IOF it provided functional information in real time and avoided the patient having to undergo further unnecessary testing.

## Case 3: Sleeve gastrectomy and achalasia

**Fig. 3:**
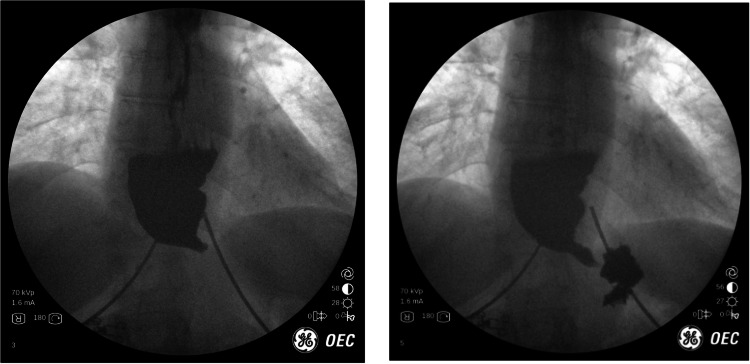
(left): contrast in dilated distal esophagus filled with saliva and secretions. (right): dilated distal esophagus with classic “bird’s beak” appearance

55 year-old female previously had a sleeve gastrectomy. She presented as a referral with a several month history of nausea, vomiting, and dysphagia. The patient described daily vomiting after meals and lost about 35 pounds over the last several months prior to presentation. The patient had seen a gastroenterologist who performed an upper endoscopy and found the esophagus to be dilated with the endoscope passing easily through the GE junction. It was interpreted as essentially normal. We performed IOF and were able to quickly understand the patient’s problem. Figure 3 shows a classic appearing dilated esophagus with “bird’s beak” appearance of the distal esophagus with minimal egress of contrast suggestive of achalasia. The image on the right shows contrast pooling in a saliva and secretion filled dilated, distal esophagus with little to no contrast emptying through the LES. Patient underwent manometry testing which confirmed Type II achalasia and they subsequently underwent a successful and uneventful robotic Heller myotomy.

The patient had complete resolution of her symptoms. This case exemplifies the importance of fluoroscopy to quickly diagnose the patient’s problem, especially when upper endoscopy or other modalities are not as useful. In this case, her symptoms were incorrectly attributed to her history of weight loss surgery, which leads to a delay in the new diagnosis of achalasia.

## Case: 4 GERD and sleeve gastrectomy with placement of magnetic sphincter augmentation

**Fig. 4:**
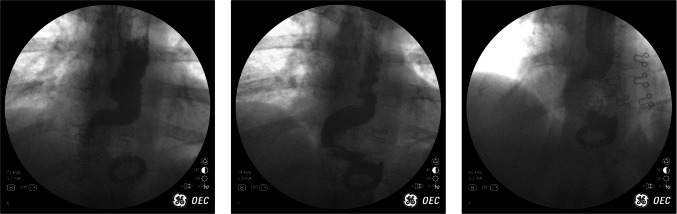
(left): LINX in good position. Significant presbyesophagus with severe esophageal dysmotility. (middle): distal esophagus makes a “C loop” with loss of peristaltic function at the mid-esophagus. (right): contrast does not move through LINX and refluxes

Reflux after sleeve gastrectomy is a well known problem [4], which can be difficult to treat. however the use of magnetic sphincter augmentation can be a solution. Here is a 44 year-old male patient with a history of a sleeve gastrectomy presented with worsening, medically refractory GERD, for which MSA was placed. He did well for several years until he developed nausea, vomiting, and abdominal pain. He presented to the ED and a CT was obtained. As the CT is a static form of imaging, it was not helpful in delineating the functionality of the MSA device during swallowing, and it did not show any abnormal anatomy as an explanation of her symptoms. She was discharged after the CT did not identify any acute issues and was told to follow up with her surgeon. We performed IOF and found severe presbyesophagus with severe esophageal dysmotility; essentially, the patient had a functional esophageal obstruction at the level of the MSA device. With IOF we rapidly diagnosed the problem and offered the solution: explantation of the device with conversion to a gastric bypass. As such, real-time functional assessment may enhance interpretation of radiologic findings in the absence of correlating clinical symptoms (Fig. 4).

This case highlights the importance of not solely relying on static imaging. Radiology reports of CT imaging can fail to identify abnormalities without the appropriate clinical context [5] and implementing fluoroscopy in real-time has allowed us to not only get to the core of the problem in the same office visit, but prevents the misdirection that usually arises from well-meaning, but inexact reports.

## Case 5: Sleeve gastrectomy and mechanical obstruction

**Fig. 5:**
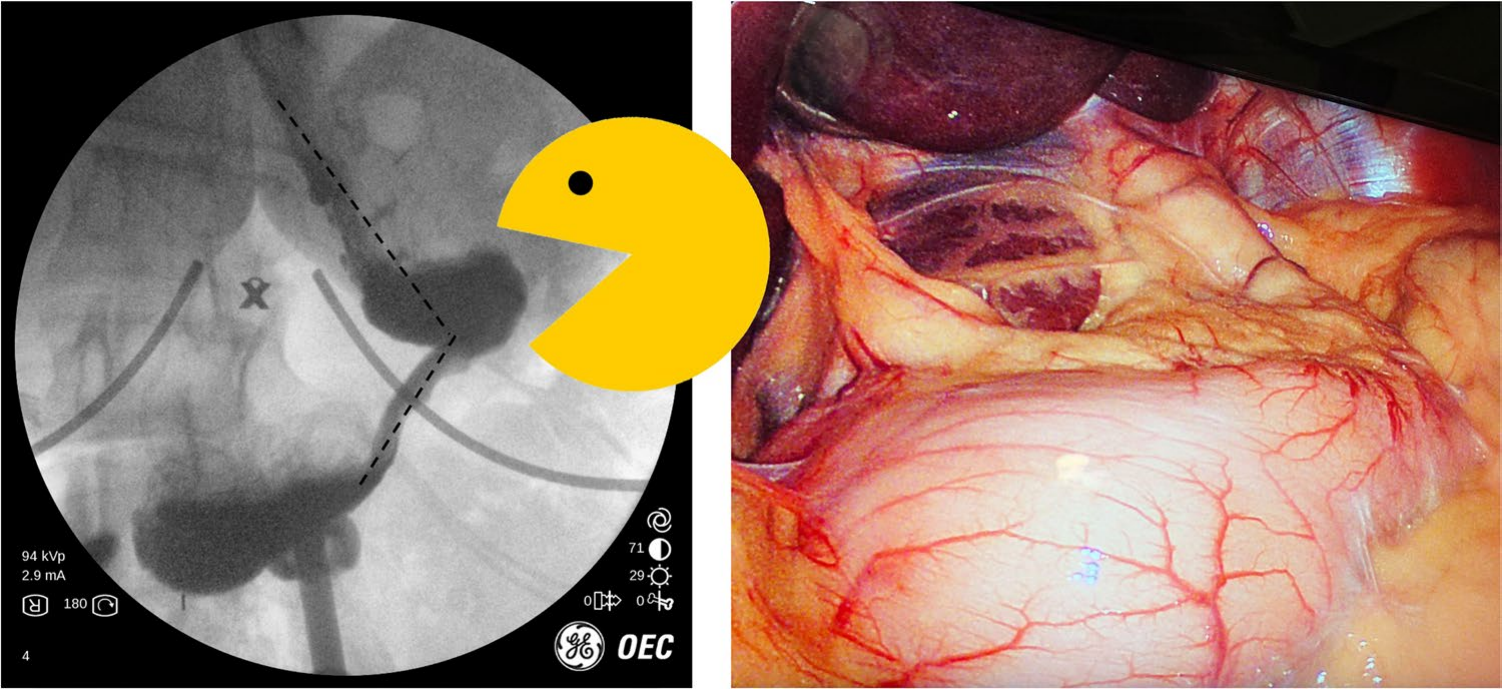
Left: “Pac-Man” sign. Contrast was noted to make its way distal to the “transition point,” but in a delayed fashion. Several episodes of reflux was noted during this study, which correlated with clinical symptoms. Right: picture at diagnostic laparoscopy. Seemingly normal appearing sleeve. However, there was kinking secondary to retrogastric adhesions along the mid-sleeve, which we released

66 year-old male with a history of sleeve gastrectomy who presented to our clinic with nausea and inability to eat solid foods. Fluoroscopy shows what we call a “Pac-Man” sign, which describes a radiographic finding that is reminiscent in appearance to the Pac-Man video game character of 1980’s fame. In effect, there is an acute angulation causing mechanical obstruction. In this instance, the sleeve develops a “transition point,” or fulcrum, beyond which contrast cannot completely empty. The buildup of pressure from secretions as well as PO intake is trapped proximally and patients complain of nausea, unrelenting reflux, and vomiting – often of white, frothy foam. Medications are usually not helpful (Fig. 5).

Intraoperatively, this can appear to be deceptively normal. Even endoscopy can be non-diagnostic, as it was in this case scenario. However, we knew from the office UGI that the patient’s sleeve was having a difficult time emptying past the fulcrum. At surgery, we found minor adhesions to what remained of the greater curve and these adhesions were taken down. It does not look problematic during laparoscopy perhaps because the stomach is not distending with a food bolus or contrast material or perhaps because they are supine. After surgery, the patient reported improvement of her daily vomiting.

Having a Pac-Man sign is an important feature to follow. It is indicative of a geometric issue that can be associated with symptoms. If a patient has this anatomic appearance associated with symptoms, surgery is often necessary.

## Case 6: Pyloromyotomy for gastroparesis

**Fig. 6:**
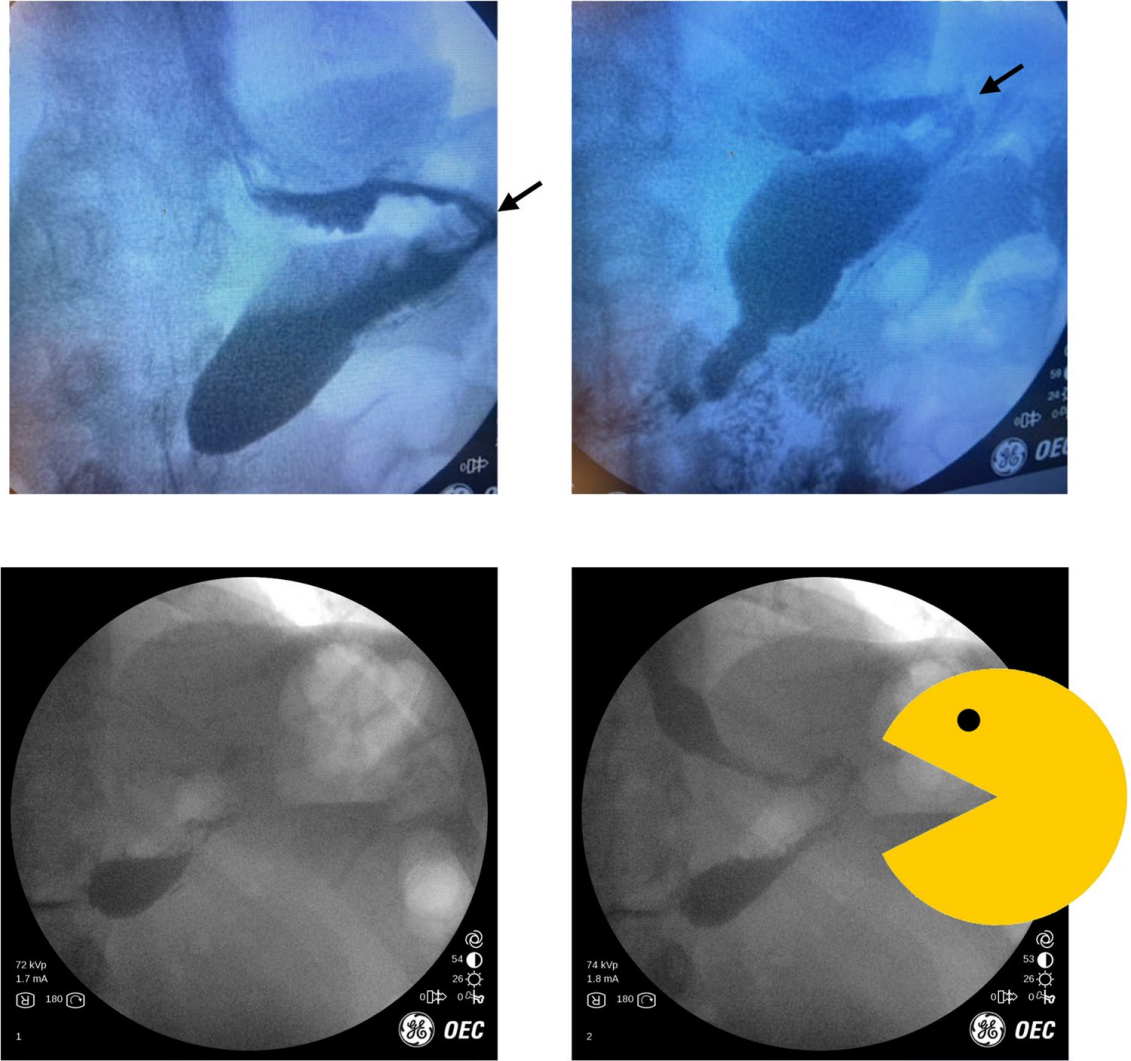
Top: UGI demonstrating gastroparesis. Bottom: After pyloromyotomy for gastroparesis in setting of sleeve gastrectomy. Fulcrum denoted by arrow

Note: although similar in appearance to the ‘Pac-man” sign described in the previous case, the findings in this are subtly different – the majority of the contrast is not retained above the fulcrum as seen in the previous case.

44 year-old female has a history of sleeve gastrectomy in 2018 complicated by several years of persistent nausea and poor PO tolerance after their surgery. The patient became malnourished with a BMI as low as 18 and was developing neurological deficits and gait instability. They underwent an upper endoscopy by one of our surgeons and the sleeve appeared normal endoscopically. However, we performed IOF and found that the sleeve was distended without passage of contrast through the pylorus for a prolonged period of time. The air-fluid levels in real-time were suggestive to our surgeons of gastroparesis. Images from the upper GI are shown in Fig. 6. The top two images show a distended sleeve with contrast sitting in the sleeve after a prolonged period of time. When the diagnosis of suspected gastroparesis was discussed with the patient, it reminded the patient that they had a history of underlying gastroparesis which was not shared with their surgeon prior to the sleeve gastrectomy. It is our practice to offer RNYGB as a treatment of gastroparesis in the patient with obesity as it offers an effective solution to both problems, rather than a sleeve gastrectomy. For this patient we performed a pyloromyotomy in an attempt to improve her PO tolerance. Postoperatively, IOF showed marked improvement in the speed of contrast out of the sleeve. In the bottom two images, one can see a less distended sleeve with contrast passing smoothly into the duodenum. The patient had immediate resolution of symptoms and now tolerates a diet without nausea or vomiting. The patient began to regain weight with improvement of neurologic symptoms.

This is not the ideal appearance or geometry for which surgeons aim. When the sleeve begins to have this deformity, it is important to solicit the patient's symptoms–if any–and their response to PPIs if indicated. Furthermore, it is important to note the clearance of contrast proximal to the fulcrum (see arrow). When contrast does not clear and is associated with symptoms, an operation is likely necessary. Being able to document these images in our medical records has been helpful to follow patients over time.

Standard gastric emptying studies may yield misleading results in patients with altered gastric anatomy such as after LSG, given rapid transit from reduced reservoir time. This may lead to a false negative result and as such, an IOF interpreted by the surgeon with understanding of the clinical context may allow for better evaluation, as in this case.

## Case 7: Roux-en-Y gastric bypass and outlet obstruction (misaligned anastomosis)

**Fig. 7:**
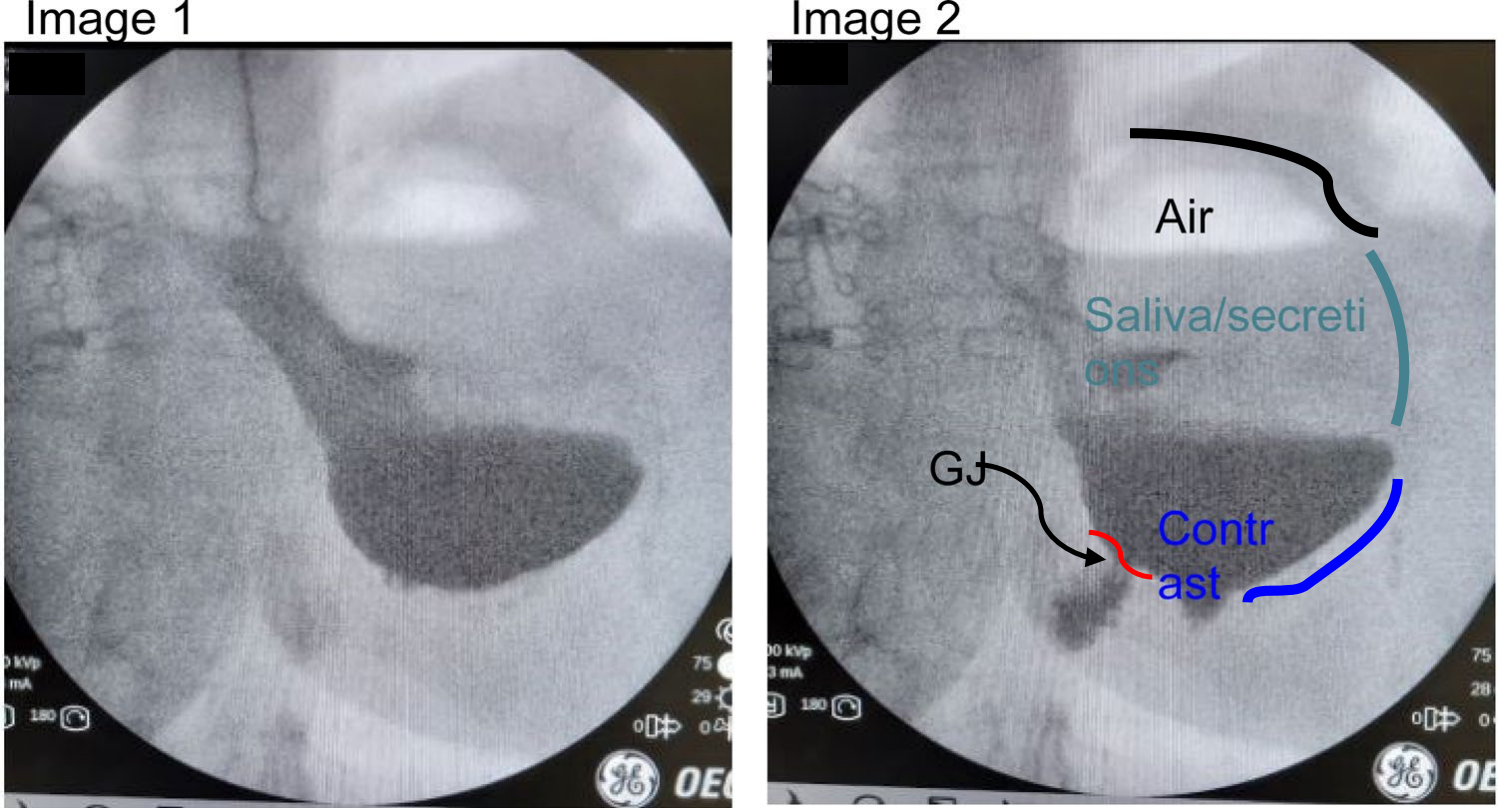
Very large gastric pouch behaving as a reservoir with a large amount of filling before any contrast is seen going into the alimentary limb (2nd image). Outflow into the alimentary limb was noted to be very slow during this dynamic study. One can appreciate the different shades of gray as diagrammed in the image: the heavier contrast material at the bottom and a lighter gray column sitting on top of this, representing saliva and secretions. Above these layers is air along the top of the gastric pouch

Our next case features a 68 year-old patient with a history of a roux-en-y gastric bypass performed 9 years prior to presenting to our clinic. The patient reported daily issues with dysphagia, heartburn, and vomiting. IOF revealed a large gastric pouch with emptying into the gastrojejunostomy only after the lateral portion of the pouch filled. Note the more dense barium fall through the less dense air and secretions to settle at the bottom of the pouch (Fig. 7).

This study demonstrates not only an enlarged gastric pouch, but also a gastrojejunostomy that is far to the right of center. We call this phenomenon “right corner pocket syndrome.” It can be seen on fluoroscopy and also on endoscopy: it refers to the location of the gastrojejunostomy when evaluating a pouch. This is extremely common and is a function of how the anastomosis was created at the time of surgery. It is not necessarily unique to a particular technique. This anatomic configuration can create, over time, preferential filling of the lateral portion of the pouch with resultant slow transit of materials through the gastrojejunostomy, despite the stoma being widely patent. The egress of contrast through the gastrojejunostomy is more of a function of hydrostatic pressure once the entire pouch is full. This geometry leads to a pouch that does not empty properly and is associated with a myriad of complaints. If an UGI is performed outside of the office without the appropriate clinical context, it could lead to a falsely normal result.

An upper endoscopy performed on this patient confirmed a 10 cm gastric pouch and a 2 cm, widely patent gastrojejunal outlet. A partial pouch gastrectomy was performed leaving the gastrojejunostomy undisturbed; essentially, a gastric pouch minimalization of the large gastric pouch. In doing so, the fundic and lateral portion of the pouch were resected and this effectively repositioned the gastrojejunostomy to the center of the bottom of the pouch.

**Fig. 8:**
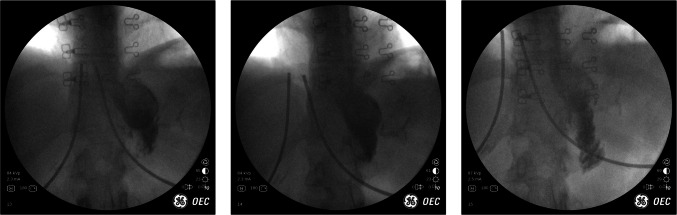
Sequential UGI images after gastric pouch revision. Contrast material draining into an appropriately sized pouch with appropriate egress through GJ outlet into the AL. No evidence of delayed emptying or reflux

The patient experienced immediate relief of symptoms initially. However, she reported experiencing some nausea and dry heaving and hiccups during subsequent follow up appointments. A repeat IOF was performed (Fig. 8).

When asked to take a sip of the contrast, the patient drank the whole cup in one swallow. This is a telltale sign that one is drinking too much and/or too quickly in the context of bariatric surgery. Therefore IOF was also used to target behavior modification. The UGI study was used to also counsel the patient on the impact that rate and volume of oral intake has on pouch emptying and how important it is to regulate these parameters. Seeing the dynamic differences in drainage in real time as it relates to oral intake helped educate the patient on the appropriate amount of food and liquids to take in with each swallow.

Concepts that were often verbalized to the patient became clearer to her when she was able watch her UGI in real-time. Many practices instruct their patients to “not eat and drink at the same time.” The UGI study can serve as a heuristic technique to understand this instruction. It helps patients to understand why they are having issues. Now, her nausea, vomiting, hiccups, and ‘reflux’ are completely resolved with the appropriate behavioral modification.

This vignette demonstrates the importance of synthesizing information from laparoscopy, endoscopy, as well as fluoroscopy in order to elucidate post-op surgical problems. The use of fluoroscopy efficiently aided in making a surgical plan to fix a problem that had been ongoing for nearly a decade. Then, it helped teach the patient how to properly use their anatomical “tool.”

**Case 8:** Roux-en-Y Gastric Bypass and Candy Cane Syndrome.

**Fig. 9:**
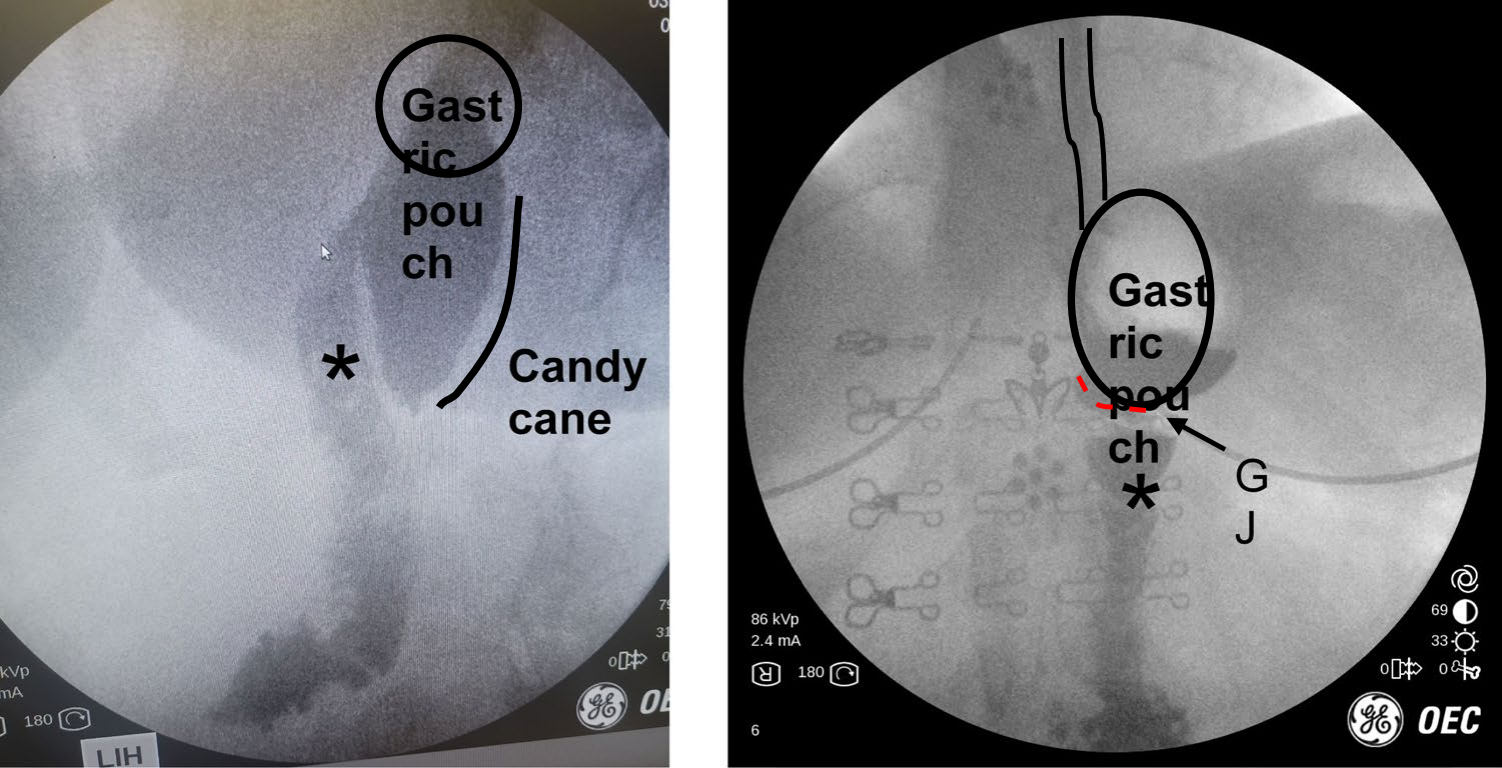
Left: pre-op UGI showing a long candy cane; there was preferential filling of contrast prior to overflowing into the alimentary limb (*). Right: post-op UGI showing smooth, and direct drainage of contrast into the alimentary limb (*)

77 year-old female developed nausea, emesis and food intolerance after undergoing a laparoscopic roux-en-y gastric bypass. The patient was admitted to an outside hospital where upper endoscopy was performed and interpreted as a possible marginal ulcer as the etiology of her symptoms. She was discharged on a PPI and instructed to follow up with our clinic. The patient continued to have daily nausea and vomiting. IOF revealed preferential filling of the blind, afferent limb of the gastrojejunostomy, a phenomenon commonly called “candy cane syndrome,” which can manifest as vomiting, pain, or reflux (see Fig. 9). The diagnosis is not based on symptoms alone: it is made as a dynamic IOF evaluation of the pouch in real time. The “candy cane,” or blind afferent limb, fills and creates extrinsic compression of the alimentary limb until there is overflow drainage of material.

Although endoscopy can also aid in the diagnosis when retained secretions or food is found in the blind afferent limb of the gastrojejunostomy, this finding can also be commonly missed, or not present on upper endoscopy or a CT scan. This is a functional problem that can often only be appreciated in a dynamic imaging setting. Treatment is resection of the excessive candy cane limb. The image on the right is our post-op IOF. The symptoms of nausea, vomiting, and abdominal pain had resolved; they are tolerating a regular diet, off PPIs, and did not have any reflux symptoms.

Performing IOF in this instance significantly shortened the time to diagnosis and intervention. Most importantly, it aided in the diagnosis and lead to the appropriate treatment of the condition.

## Case 9: Roux-en-Y Gastric Bypass and Gastric Outlet Obstruction (stricture)

56 year old male with a history of a roux-en-y gastric bypass done in 2008 presented to our clinic with nausea and vomiting. The patient underwent an upper endoscopy which confirmed a stricture of the gastrojejunostomy and underwent balloon dilation and stent placement by gastroenterology. It was not effective in treating his symptoms.

**Fig. 10:**
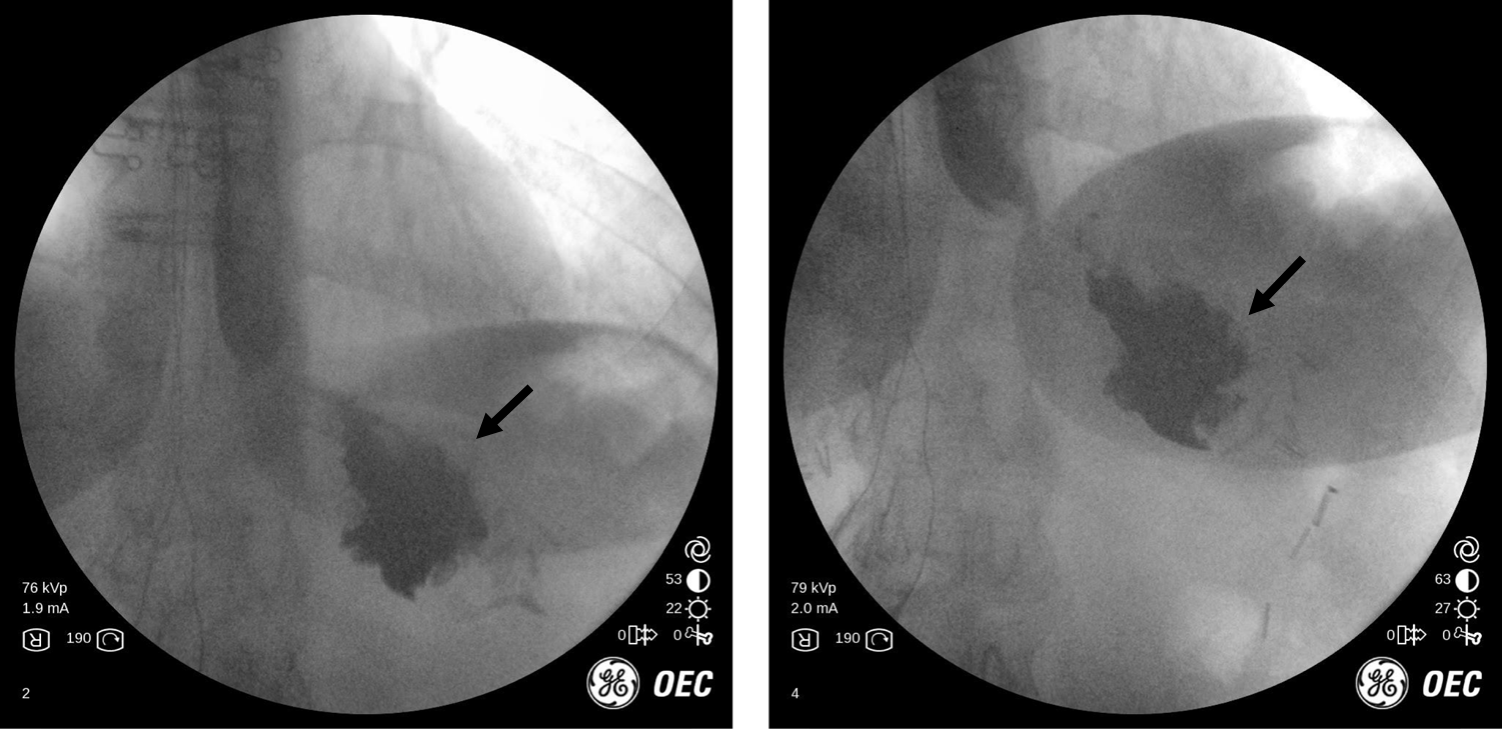
UGI study from office revealing failure of contrast to progress beyond gastric pouch. Several minutes have passed between the images

The patient underwent IOF and was found to have an outlet problem of the gastrojejunostomy. Images from the upper GI are shown in Fig. 10. In the image to the left, contrast is seen in the esophagus and entering the gastric pouch however there is no contrast emptying out of the pouch concerning for outlet obstruction. The non-uniform appearance of the pouch is indicative of food debris still present in the pouch. After several minutes, repeat image on the right shows contrast that has failed to empty out of the pouch.

The patient underwent gastrojejunostomy revision and his symptoms resolved. IOF in this scenario served a complementary role in the diagnosis, as the upper endoscopy did already confirm the stricture.

## Case 10: Sleeve Gastrectomy and Retained Fundus and Subsequent Achalasia

46 year-old female with a remote history of a sleeve gastrectomy with hiatal hernia repair presents with dysphagia, nausea, vomiting, and weight regain. IOF done revealed a significant amount of retained fundus, and interpretation of the images in real-time did not reveal any concern for esophageal dysmotility, and it was interpreted that the large retained fundus with pooling on contrast on IOF was the cause of her nausea and vomiting. A diagnostic laparoscopy was hence performed and decision was made to resect the retained fundus. Operative intervention coupled with behavioral modification improved the patient’s symptoms. Her nausea and dysphagia had resolved and She began to lose weight.

**Fig. 11:**
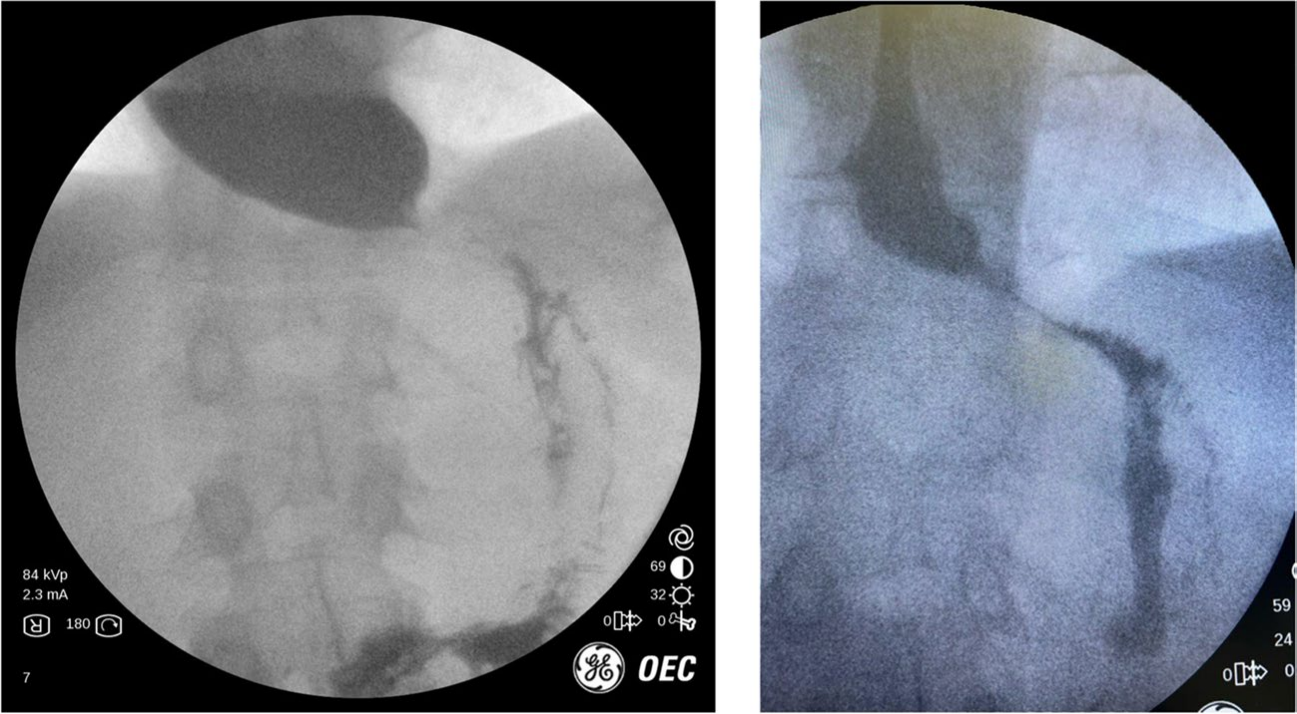
Left: pre-op UGI revealing a bird’s beak sign characteristic of achalasia. Egress of contrast into sleeve was very delayed. Right: after Heller myotomy. Rapid transit of contrast from esophagus into sleeve was noted

A year later the patient presents with symptoms of dysphagia, nausea, vomiting, and reflux. Another IOF was performed (see Fig. 11) which revealed that the distal esophagus was dilated and there was very little contrast exiting into the sleeve. This was not seen on previous IOF images. Upper endoscopy did not reveal any hiatal hernia or significant stenosis of the GEJ. A manometry study was obtained which revealed high LES basal pressure and ineffective swallows 94% of the time, confirming the diagnosis of achalasia. A Heller myotomy was performed. The patient did well and her symptoms resolved. IOF at 6 months demonstrated rapid transit of contrast through the GEJ into the sleeve without any evidence of reflux.

## Case 11: Sleeve Gastrectomy and Delayed Gastric Emptying

**Fig. 12:**
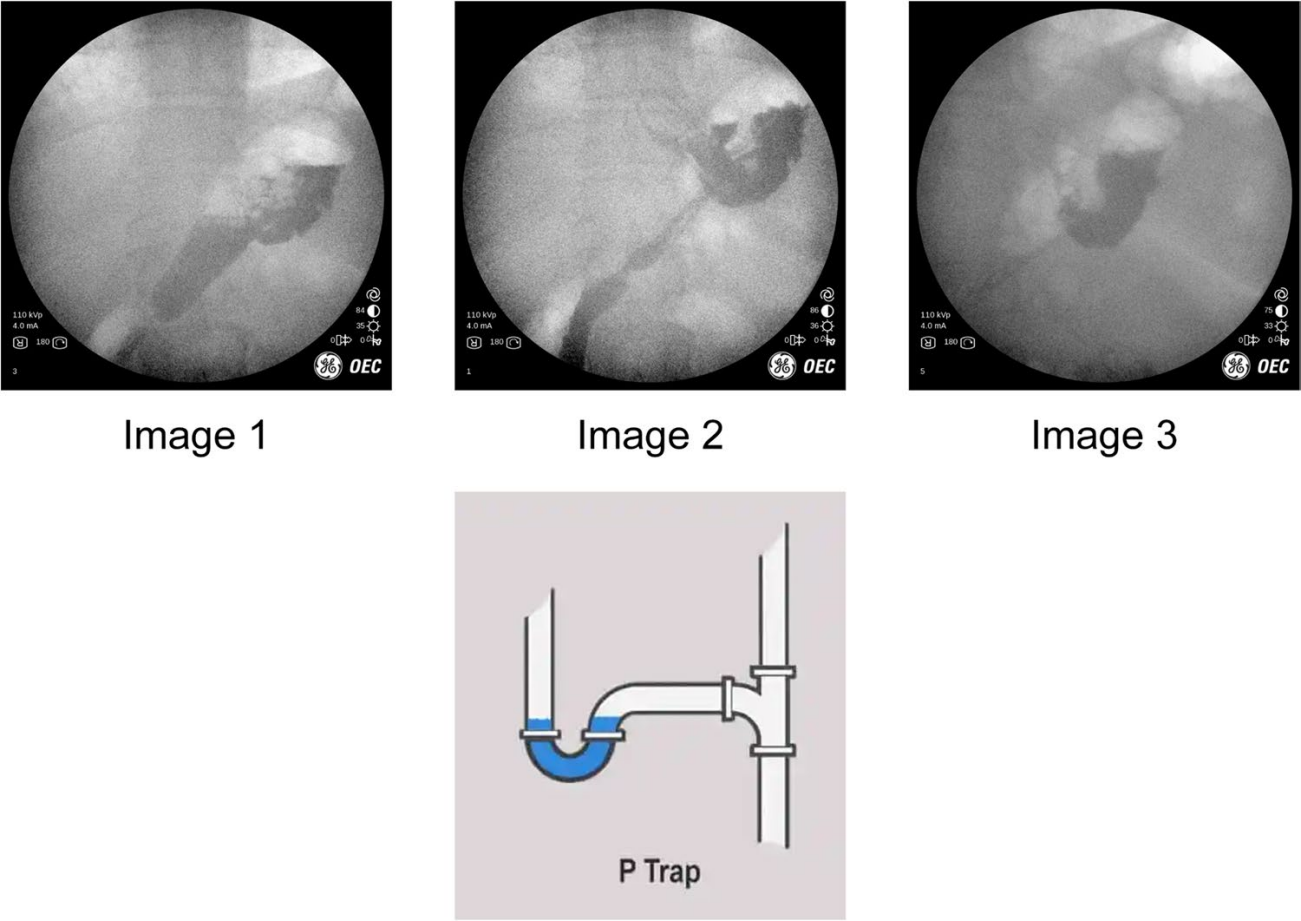
IOF of angulated sleeve resembles a 'p-trap'

43 year-old female underwent a sleeve gastrectomy in 2013. The patient did not follow-up for 9 years, then presented with a 6 month history of reflux, nausea, and vomiting. The patient described symptoms to be worse at night while laying down. He was refractory to antacids. IOF demonstrated several issues with the geometry of her sleeve. Images obtained are in Fig. 12: the sleeve is not dilated and does empty through the pylorus. However, the issue is that contrast is retained where the sleeve is angulated similar to how a ‘p-trap’ traps water in plumbing. In the patient’s sleeve, however, the chronically trapped fluid was likely a constant source of nausea and reflux.

The first image shows contrast going through the sleeve and emptying normally through the pylorus. Subsequent images show how contrast remains in the “p-trap” despite the rest of the contrast egressing normally. The third image is a closer view of the contrast that is not able to drain because it is stuck in the trap. One can appreciate the empty distal sleeve in the background. Note: when patients do IOF they are upright. This allows us to redemonstrate issues that cannot be appreciated when the patient is supine, as on the OR table during a diagnostic laparoscopy for example.

Contrast – and thus food and secretions– does not empty well in this scenario. In these cases, patientl complain of reflux, nausea, and occasionally vomiting. There is often no esophageal dysmotility or dilation. In these instances there is a conformational change in the geometry of the sleeve whereby the outflow or emptying of the sleeve is greatly impacted. The proximal sleeve fills and then takes a “p-trap” appearance, increasing the amount of hydrostatic pressure proximally in the sleeve, which explains the symptoms of reflux and vomiting. It is the material above the fulcrum that never completely clears until it is displaced by secretions. It is unclear to our practice why this happens. But when it does, in our experience conversion to a RNYGB is often necessary for symptomatic relief. Note: We have attempted to perform gastroplasty to straighten out the sleeve, however, outcomes on several patients have not been favorable. There are often few findings as the sleeve looks normal during diagnostic laparoscopy and intraoperative EGD. CT scan is often not helpful in this scenario [6]. IOF is an important diagnostic modality of this phenomenon.

## Discussion

We have found doing IOF to be efficient and useful in our practice. By offering this in the office setting, we have appreciated the following:

- Increased efficiency: IOF provides a minimum of two fewer clinic appointments for patients. Patients no longer need to go to an imaging center and as a result eliminate the need for another follow up appointment to discuss results from where the patient was referred. As a result, patients miss fewer days of work as well as eliminate costs from an imaging center. IOF often simplifies the investigative process because many important imaging findings can be missed without the appropriate clinical context the surgeon has. IOF performed by the treating surgeon provides invaluable, real-time, dynamic evaluation of the anatomy in a video format instead of mostly static images taken by a technician or radiologist who does not fully comprehend the entire clinical picture.
- Behavior modification and decision making: Using fluoroscopy as a behavior modification tool is especially useful in patients with remote histories of weight loss surgery. For example, it is not uncommon to see a patient who had a sleeve gastrectomy many years prior who is experiencing weight recurrence, reflux, or hiccups. The combination of these three issues usually suggests the patient is eating too much or too quickly, or both. We have found that hiccups and/or reflux are a common manifestation of liquid or food backing up proximally due to the pylorus naturally inhibiting outflow. This resultant delay in gastric emptying is desirable insofar as weight loss is concerned as it increases both satiety and satiation. Once we use fluoroscopy to demonstrate how certain choices are contributing to problems, the insight invariably leads to improvement in symptoms simply by virtue of facilitating a change in the patient’s eating habits. IOF also aids in decision making: a patient can have issues with their sleeve or pouch geometry and shape that appears normal on endoscopy. These patients often have bounced from clinician to clinician and they are understandably frustrated. IOF, however, provides another more dynamic and real-time way to assess patients and it can accelerate the ability to make a decision for surgery, if indicated.
- Dealing with surgical tourism: Utilizing IOF is helpful in diagnosing and treating patients that have participated in surgical tourism. With our proximity to Mexico, we've had multiple patients come to us to establish care only to discover that they did not have the operation that they paid for. We have seen patients who have undergone sham bariatric operations such as patients with only the subcutaneous port of a gastric band apparatus implanted; patients who were told they had a gastric bypass only to discover they simply had a Penrose drain tied around the mid-body of the stomach. One can only imagine how upsetting this would be to discover, however, it is important to know that any attempts at endoscopy or a CT scan would likely not have been diagnostic.
- Completeness of care for foregut surgery patients: We have been able to utilize fluoroscopy for evaluating patients with paraesophageal hernias, achalasia, and other motility issues both in the preoperative and postoperative setting. Our practice offers treatment options of certain reflux conditions with magnetic sphincter augmentation devices and with the use of fluoroscopy, patients are able to easily see how their device works when they watch themselves swallow in real time.
- We hope that these vignettes will inspire surgeons to incorporate IOF into their clinical practice. There are a few obstacles that might present but overall this not difficult to implement. For example, different countries may have their own radiology regulations. In the US, each state has their own requirements. In Texas, we have a requirement for our office to maintain a Radiation Protocol Committee (RPC) within our staff to establish our fluoroscopy protocols and meet with state physicists annually to review those protocols. In addition, our providers are required to have completed an 8 h Physician Awareness Training. The clinic also is required to maintain a method used to monitor radiation exposure. In our case, our fluoroscopy equipment generates this value for us based on fluoroscopy time and we are required to monitor the dose of radiation exposure for each patient. The RPC within our staff is required to establish protocols for actions to be taken if the dose of radiation exposure for a patient exceeds the established reference levels, such as recommending an interval of follow-up appointment for monitoring symptoms.
- Limitations: There are some limitations our practice acknowledges in the diagnostic utility of IOF.

The skill and experience of the surgeon performing the IOF play a crucial role in image acquisition and interpretation. Factors like patient positioning, instructions on volume and timing of contrast swallows, and the interpretation of images can vary between surgeons. In particular, interpretation of IOF images can be subjective and influenced by the operator's experience and knowledge.

This educational review is a series of patient case vignettes encountered in a single center, and as such can arguably be limited in terms of its generalizability.

Although minimal compared to many conventional diagnostic modalities like CT scans, the use of IOF does expose the patient as well as the operator to small amounts of radiation. This exposure risk is further reduced when appropriate radiology regulations, protocols and protective equipment are in place.

## Conclusions

In-office fluoroscopy provides bariatric and foregut surgeons a dynamic, highly informative diagnostic tool that enhances clinical decision-making and patient education. We advocate for its broader incorporation into routine surgical practice.

## Data Availability

No datasets were generated or analysed during the current study.
